# The prognostic value of the Frank sign

**DOI:** 10.1007/s12024-022-00463-8

**Published:** 2022-02-18

**Authors:** J. Prangenberg, E. Doberentz, L. Johann, B. Madea

**Affiliations:** 1 Institute of Legal Medicine, University Hospital Bonn, Stiftsplatz 12, 53111 Bonn, Germany; 2 Department of Forensic Medicine, University of Göttingen, Robert-Koch-Straße 40, 37075 Göttingen, Germany

**Keywords:** Atherosclerosis, Autopsy study, Coronary artery disease, Diagonal earlobe crease, Frank’s sign

## Abstract

Frank’s sign (named after American pulmonologist Sanders T. Frank) refers to a diagonal skin fold between the tragus and outer edge of the earlobe. Gradation is based on the bilateral presence and/or degree of the earlobe fold. The presence of this sign, referred to as the diagonal earlobe crease (DELC), has been associated with coronary artery disease (CAD), independent of other cardiovascular risk factors. Corresponding studies are predominantly based on clinical or angiographic assessments, and few autopsy studies exist. The association of DELC with CAD, cardiovascular risk factors, and causes of death was investigated via retrospective and prospective evaluations. It was also investigated whether the degree of DELC correlated with the macroscopic severity of coronary heart disease. Furthermore, the influence of age on the appearance of DELC was analyzed and compared using two age groups. Additionally, binomial logistic regression analysis was performed to investigate the influence of age on the presence of higher-grade DELC and CAD. In cases related to a lethal cardiac event, the majority (78%) showed high-grade DELC. The DELC grade correlated significantly with CAD severity (*r*_*s*_ = 0.474, *p* < 0.001) and with the severity of general atherosclerosis (*r*_*s*_ = 0.606, *p* < 0.001) with medium and large effects sizes, respectively. Age was predominantly more suitable than DELC concerning the sensitivity, specificity, and positive prognostic value for preexisting cardiac disease and cardiac-related causes of death. In both DELC and CAD, age has a significant influence on the presence of higher-grade manifestation, but the influence of age in CAD appears to be even more significant than in DELC. The main results of previous autopsy studies and the prognostic value could have been confirmed, but these findings appear to be limited to younger patients.

## Introduction

**Fig. 1:**
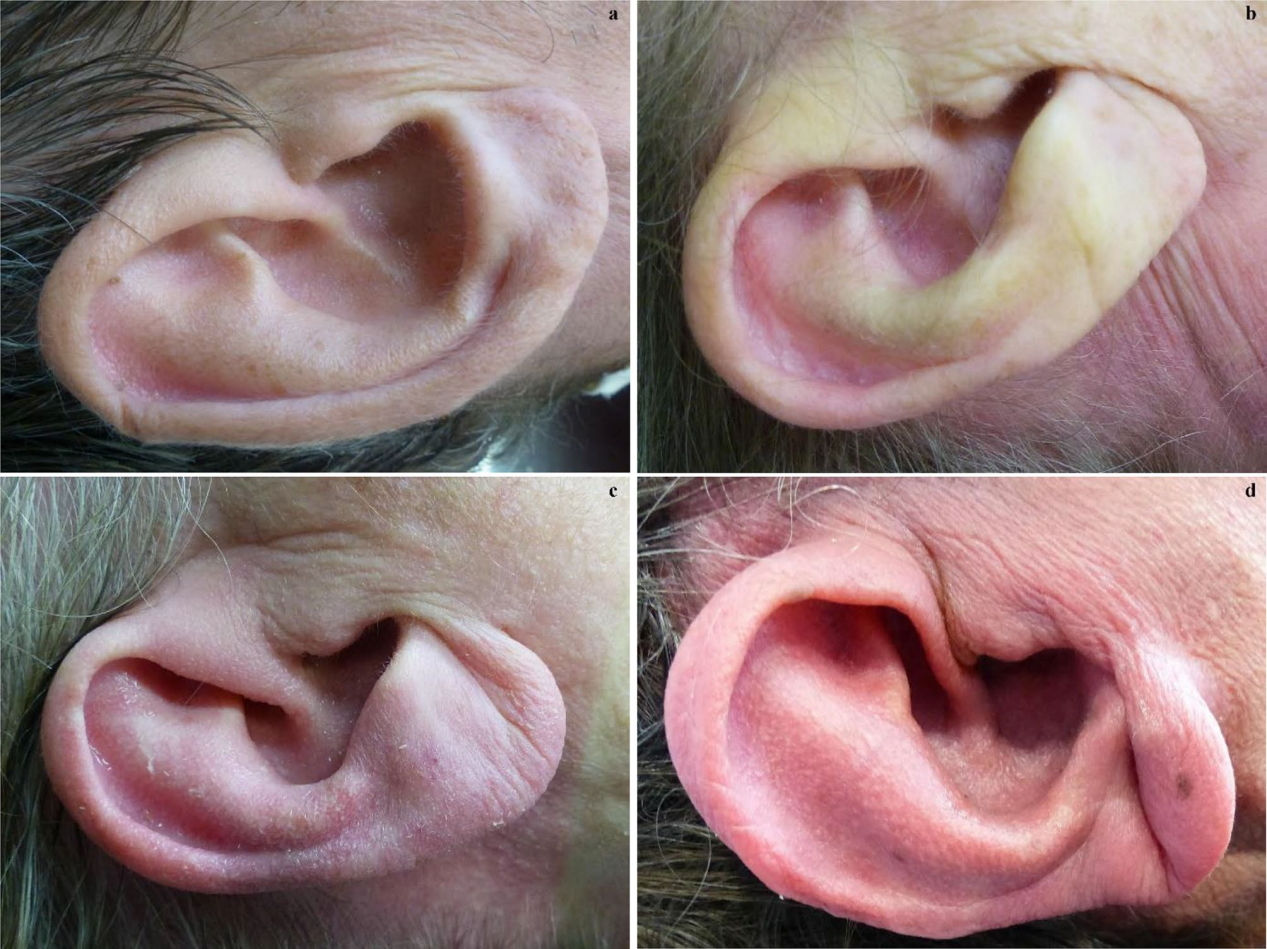
DELC grading

The Frank sign was first established in 1973 by American pulmonologist Sanders T. Frank to describe a unilateral or bilateral diagonal fold of skin between the tragus and outer edge of the earlobe, which is also called a diagonal earlobe crease (DELC) [1]. Gradation may be based on the bilateral occurrence and/or degree of the earlobe crease (Fig. 1). Grade 1 is a slight wrinkling of the skin around the earlobe. Grade 2a involves a superficial skin fold that partially covers the earlobe at least halfway; grade 2b involves a superficial skin fold that covers the earlobe completely. Grade 3 involves a deep skin fold that covers the entire earlobe [2]. Frank described a correlation between the occurrence of this skin fold and the presence of coronary heart disease [1]. This correlation is assumed to exist because both the earlobe and heart are supplied by “end arteries” and therefore lack collateral circulation. Another assumption is that the general loss of elastin and elastic fibers observed in biopsy specimens from the earlobes reflects the presence of microvascular disease in the coronary arteries [3–5].

Since its first description by Frank, various studies have confirmed the association between a DELC and coronary heart disease [6–12] as well as an association with increased cardiac morbidity and mortality [13]. In 1996, Elliott and Powell postulated a relative risk for a cardiac event for a unilateral earlobe crease of 1.33 and a bilateral earlobe crease of 1.77 [14]. The occurrence of a DELC is reported be more common in patients with myocardial infarction than in patients without myocardial infarction [15, 16]. However, the occurrence of a DELC in association with coronary artery disease (CAD) has been reported to be influenced by the individual’s age, as noted by Mehta and Hamby in 1974 [17]. Since then, several studies have denied the association between a DELC and atherosclerotic disease, proposing that it is simply a phenomenon of age [18–20].

An important consideration is that these studies were based mainly on clinical or angiographic assessments, and few autopsy studies have been conducted [2, 21–24]. In this study, the associations between the degree of Frank’s sign with cardiovascular diseases and cardiac-related causes of death in the context of forensic autopsies were investigated, and their prognostic values were compared based on the age of the deceased.

## Materials and methods

We conducted retrospective and prospective evaluations of photographic material obtained from the Institute of Legal Medicine of the University Hospital Bonn between January 2015 and April 2020. Inclusion criteria included cases that had clearly assessable images of at least one earlobe. Exclusion criteria were cases with putrefaction, anasarca, the presence of earrings, and/or a generally strong skin wrinkling of the head and facial regions.

Macroscopically visible alterations of the coronary and main arteries of the body on postmortem examination were estimated and classified into four grades: grade 1: fatty deposits in the vessels without calcification; grade 2: calcification without stenosis; grade 3: calcification with moderate stenosis (< 70% in a major coronary vessel or main artery); and grade 4: calcification with significant (> 70% in a major coronary vessel or main artery) or complete stenosis. A preexisting cardiac disease was assumed when a CAD grade ≥ 3, ventricular hypertrophy, cardiomyopathies, and/or previous myocardial infarction was present.

To determine the prognostic value of Frank’s sign, we divided the collective into two groups with either low grade (grade up to 2a) or high grade DELC (grade higher than 2b) and carried out Student’s *t*-tests for two independent samples using a 95% confidence interval. In the next step, we divided the collective into two groups based on their age to evaluate the influence on the appearance of the DELC. Based on the median age calculated, we formed one group with age < 63 years and the other with age ≥ 63 years. We also tested the correlation between the DELC grade and CAD degree, atherosclerosis, heart weight, visible preexisting cardiac diseases during autopsy, and cardiac-related cause of death by using Spearman’s rank correlation coefficient and Cohen’s effect sizes (0.10 = small, 0.30 = medium, 0.50 = large). In addition, we calculated the sensitivity, specificity, positive predictive value (PPV), and negative predictive value (NPV) for the DELC, and assessed the age of diagnosis of preexisting cardiac disease and cardiac-related cause of death. To examine the influence of age on the presence of higher-grade DELC (above 2b) and CAD (above 2), a binomial logistic regression analysis was performed. Prior to this analysis, the study population was divided into four approximately equal age groups: 1 to 47 years (*n* = 39), 49 to 62 years (*n* = 42), 63 to 75 years (*n* = 43), and 76 to 96 years (*n* = 41). The age group first mentioned served as a reference.

## Results

The study collective comprised 165 deceased persons (115 male, 50 female) with an mean age of 61 years (range 11–96 years, median 63 years, SD 19.01), of which 27 persons (16.3%) had no visible DELC, 12 (7.3%) showed DELC grade 1, 18 showed DELC grade 2a, 53 (32.1%) showed DELC grade 2b, and 55 (33.3%) showed DELC grade 3.

Tables 1 and 2 show the statistical analyses of the two groups divided by the DELC grade and age. Deceased persons with a DELC grade above 2b were significantly older, had a higher heart weight, a higher grade of CAD severity, and atherosclerosis; frequently, they had a preexisting cardiac disease and a cardiac-related cause of death. In comparison, deceased persons older than 63 years of age showed statistically significant differences in the abovementioned variables. Furthermore, the DELC and age showed predominantly medium and large effect sizes, respectively (Table 3).

**Table 1:** Comparison of the collective divided by DELC grade

**Variable**	**DELC grade**	**n**	**Mean value**	**Standard deviation**	***p*-value**
Age (years)	Grade ≤ 2a	57	46.75	18.65	< 0.001
	Grade ≥ 2b	108	68.40	14.45	
Heart weight (g)	Grade ≤ 2a	57	402.02	111.70	< 0.001
	Grade ≥ 2b	108	490.14	128.75	
CAD (grades 0 to 4)	Grade ≤ 2a	57	1.75	1.02	< 0.001
	Grade ≥ 2b	108	2.57	1.11	
Atherosclerosis (grades 0 to 4)	Grade ≤ 2a	57	1.37	0.70	< 0.001
	Grade ≥ 2b	108	2.29	0.93	
Preexisting cardiac disease (0 = no, 1 = yes)	Grade ≤ 2a	57	0.26	0.44	0.002
	Grade ≥ 2b	108	0.52	0.50	
Cardiac-related cause of death (0 = no, 1 = yes)	Grade ≤ 2a	57	0.18	0.38	0.071
	Grade ≥ 2b	108	0.31	0.46

**Table 2:** Comparison of the collective divided by age

**Variable**	**Age (years)**	***n***	**Mean value**	**Standard deviation**	***p*-value**
DELC (grades 0 to 4)	< 63	81	1.79	1.50	< 0.001
	≥ 63	84	3.36	0.82	
Heart weight (g)	< 63	81	425.31	129.93	0.001
	≥ 63	84	492.86	121.41	
CAD (grades 0 to 4)	< 63	81	1.74	0.98	< 0.001
	≥ 63	84	2.82	1.04	
Atherosclerosis (grades 0 to 4)	< 63	81	1.33	0.59	< 0.001
	≥ 63	84	2.58	0.84	
Preexisting cardiac disease (0 = no, 1 = yes)	< 63	81	0.25	0.43	< 0.001
	≥ 63	84	0.61	0.49	
Cardiac-related cause of death (0 = no, 1 = yes)	< 63	81	0.14	0.34	< 0.001
	≥ 63	84	0.38	0.49

**Table 3:** Spearman’s correlation and Cohen’s effect size

**DELC**	***p*-value**	**Effect size**
Age	< 0.001	0.67
Heart weight	< 0.001	0.42
CAD	< 0.001	0.47
Atherosclerosis	< 0.001	0.61
Preexisting cardiac disease	< 0.001	0.44
Cardiac-related cause of death	0.001	0.26
**Age**	***p*-value**	**Effect size**
DELC	< 0.001	0.67
Heart weight	< 0.001	0.37
CAD	< 0.001	0.57
Atherosclerosis	< 0.001	0.74
Preexisting cardiac disease	< 0.001	0.50
Cardiac-related cause of death	< 0.001	0.29

In 50 of the 165 cases, death was directly or at least partially related to a cardiac event. Regarding those 50 cases, 11 decedents (22%) had no or only low-grade DELC, whereas 39 (78%) had high-grade DELC (grade 2b or 3). In comparison, 14 of the decedents (28%) were younger than 63 years, whereas 36 (72%) were older. The diagnostic values for DELCs and age regarding preexisting cardiac disease and cardiac-related causes of death are displayed in Table 4. The DELC showed a higher sensitivity but a lower specificity for the diagnosis of a preexisting cardiac disease and cardiac-related cause of death compared to age. The DELC and age differed slightly only for the PPV and NPV for these two variables. The binomial logistic regression model was statistically significant for presence of higher-grade DELC (*χ*^2^(3) = 42.02, *p* < 0.001) and CAD (*χ*^2^(3) = 49.84, *p* < 0.001), resulting in an acceptable amount of explained variance (Nagelkerke’s *R*^2^ = 0.0309 and 0.351, respectively). Age contributed significantly in predicting the presence of higher-grade DELC as well as CAD (*p* < 0.001, each). The summary of odds ratios for different age groups is shown in Table 5.

**Table 4:** Diagnostic values of the analyzed variables

**Variable**	**Sensitivity (%)**	**Specificity (%)**	**PPV (%)**	**NPV (%)**
Preexisting cardiac disease (DELC)	79.5	51.2	62.3	71.2
Preexisting cardiac disease (age)	69.9	73.2	72.5	70.6
Cardiac-related cause of death (DELC)	78.0	40.9	36.4	81.0
Cardiac-related cause of death (age)	72.0	60.9	44.4	83.3

*PPV* positive predictive value, *NPV* negative predictive value

**Table 5:** Summary of odds ratios for different age groups

	*p*	Odds ratio	95% Cl for odds ratio	
			Lower bound	Upper bound
**DELC above grade 2b**				
49 to 62 years	0.001	4.712	1.821	12.198
63 to 75 years	> 0.001	12.687	4.431	36.326
76 to 96 years	> .001	20.880	6.418	67.929
**CAD above grade 2**				
49 to 62 years	.008	8.293	1.732	39.705
63 to 75 years	> .001	23.368	4.985	109.542
76 to 96 years	> .001	50.455	10.375	245.375

Reference group: 1 to 47 years

## Discussion

The result of this study confirms the association between the occurrence of a DELC and the presence of CAD and general atherosclerosis, as well as a correlation between the respective grades of severity. The DELC was present with a frequency similar to that observed in the autopsy study by Kirkham et al. (72% vs. 84%), yet a cardiovascular cause of death was significantly more frequent in that study (64% vs. 30%) [24]. On the one hand, this may be because the collective age of the study cohort was 10 years older, on average. On the other hand, a substantial proportion of the retrospectively examined collective included cases of deaths due to hanging and strangulation. Cumberland et al. included 800 consecutive autopsies with an average age of 34.8 years. The youngest patient to be identified with an earlobe crease was 27 years of age. It was postulated that there was a statistically significant correlation between the DELC presence and severe stenotic coronary atherosclerosis [21]. In contrast with our study, Cumberland et al. did not grade the DELC, so it was unclear at what severity the DELC presence was affirmed.

In both higher-grade DELC and CAD, age had a significant effect on its presence. However, it was striking that the association between the presence of higher-grade CAD was more than two times higher in the different age groups than in higher-grade DELC. This suggests that although the presence of DELC, like CAD, is age-dependent, age itself has a comparatively significantly higher influence in the presence of CAD.

The sensitivity, specificity, PPV, and NPV for DELCs with a CAD diagnosis were 75.2%, 53.8%, 67.1%, and 63.3%, according to a study of 449 in-hospital cases by Wu et al. [8]. Based on 520 forensic autopsy cases, Edston calculated a sensitivity of 75% and a PPV of 68% for DELC with a CAD diagnosis. The highest PPV was observed in the group of persons aged younger than 40 years [22]. The results of those studies are similar to our calculated DELC values with the diagnosis of a preexisting cardiac disease. Cumberland et al. [21] calculated a sensitivity of 55%, specificity of 83%, PPV of 42%, and NPV of 90% for the DELC and concomitant significant atherosclerosis, and, consequently, a much lower sensitivity as well as a much higher specificity and NPV than those of our findings.

.An autopsy study by Stoyanov et al. which was one of the rare studies that included histological investigations, showed a significant association between the DELC and histopathological changes in the myocardium [25]. In accordance with our results, they observed a significant association between increased heart weight and the presence of a DELC. Furthermore, and in accordance with the results of studies by Patel et al. [2] and Ishii et al. [23], a more severe degree of CAD can be expected in cases of high-grade DELCs. A compilation of these and previously performed autopsy studies is summarized in Table 6.

**Table 6:** Summary of autopsy studies on DELC

**Study**	**Year**	***n***	**Age (overall)**	**Sensitivity (%)**	**Specificity (%)**	**PPV (%)**	**NPV (%)**	**General findings**
Cumberland et al.	1987	800	Age ranged from 1 1/2 months to 87 years. Mean age 34	55^a^	83^a^	42^a^	90^a^	Positive correlation between the presence of the diagonal earlobe crease and obstructive coronary atherosclerosis. Youngest patient with an earlobe crease was 27 years old
Kirkham et al.	1989	303	Mean age 72 ± 15	n.p	n.p	n.p	n.p	Diagonal earlobe creases are associated with cardiovascular causes of death
Ishii et al.	1990	134	Age ranged from 3 to 87 years. Only males	n.p	n.p	n.p	n.p	Earlobe crease was dependent on the extent of coronary and aortic atherosclerosis, but was independent of age. No subjects under the age of 40 were observed to have earlobe crease
Patel et al.	1992	376	Mean age 70 ± 11 (m) and 77 ± 11 (f). All persons were older than 40 years	62 (m ^b^/69 (f)^b^	66 (m)^b^/78 (f)^b^	n.p	n.p	Risk of death from myocardial infarction was 2.50 in men with high-grade creases and 3.70 in women
Edston et al.	2006	520	Age ranged from 16 to 95 years. Mean age 56 ± 18	75^c^	64^c^	68^c^	72^c^	ELC was found to be the strongest independent risk factor for CAD and SCD apart from age and BMI (both genders). Diabetes mellitus was not associated with ELC
Wu et al.	2014	449	Mean age 63 ± 12	75^d^	54^d^	67^d^	63^d^	Significant association between DELC and CAD independent of established risk factors in Chinese population
Stoyanov et al.	2021	45	Age ranged from 23 to 88 years. Mean age 64 ± 13	n.p	n.p	n.p	n.p	Data suggests a significant correlation between the morphological changes of the myocardium and the presence of the ear lobe creases
Prangenberg et al.	2021	165	Age ranged from 11 to 96 years. Mean age 61 ± 19	79^e^/78^f^	51^e^/41^f^	62^e^/36^f^	71^e^ /81^f^	DELC grade correlated significantly with CAD severity and with the severity of general atherosclerosis. In both DELC and CAD, age has a significant influence on the presence of higher-grade manifestation, but the influence of age in CAD appears to be even more significant than in DELC

*n.p.* not presented

^a^For DELC and concomitant significant atherosclerosis

^b^for detecting severe coronary atheroma

^c^for the severity of CAD expressed as CAD score

^d^for DELC to predict CAD

^e^for DELC and preexisting cardiac disease

^f^for cardiac-related cause of death

In conclusion, the main results of previous autopsy studies and, thus, the prognostic value and clinical relevance of the DELC could have been confirmed. Compared to age, the DELC showed a higher sensitivity (79.5% vs. 69.6%) but lower specificity (51.2% vs. 73.2%) and lower PPV (62.3% vs. 72.5%) for preexisting cardiac disease. Its specificity (40.9% vs. 60.9%) and PPV (36.4% vs. 44.4%) were also lower for cardiac-related causes of death.

## Conclusion

The DELC and age showed a statistically significant correlation between age, heart weight, CAD, general atherosclerosis, preexisting cardiac disease, and cardiac-related causes of death. Consequently, the occurrence of a DELC must be considered in the context of the individual’s age. In both DELC and CAD, age has a significant influence on the presence of higher-grade manifestation, but the influence of age in CAD appears to be even more significant than in DELC. Therefore, the presence of a DELC seems to be of prognostic value for younger patients especially, and it seems advisable that its presence should be inspected regularly during physical examination. As recently emphasized by Stoyanov et al. [26], a large-scale prospective cohort study is needed to clarify the correlation between DELC and cardiovascular morbidity and its role as a marker for related complications. Such a study, which should also include a sufficiently large number of young subjects, would certainly also be suitable to clarify the influence of age and the correlation between DELC and CAD.

Furthermore, descriptions of the nomenclature and classification of Frank’s sign were inconsistent throughout the different studies. As mentioned earlier, the DELC grade may be based on the bilateral occurrence and/or the degree of the earlobe crease, which can only be affirmed by its presence. A coherent definition of this sign, with respect to the requirements needed for diagnosis, would greatly increase the comparability of the studies conducted on this subject and would help to clarify its prognostic value for cardiovascular and other medical conditions.

## Key points

1. Frank’s sign refers to a diagonal earlobe crease (DELC).
2. The presence of Frank’s sign has been associated with coronary artery disease.
3. In cases related to a lethal cardiac event, the majority showed high-grade DELC.
4. DELC grade correlated significantly with severity of CAD and general atherosclerosis.
5. Age has a significant influence on the presence of higher-grade DELC and CAD.
